# Circulating Docosahexaenoic Acid Associates with Insulin-Dependent Skeletal Muscle and Whole Body Glucose Uptake in Older Women Born from Normal Weight Mothers

**DOI:** 10.3390/nu9020110

**Published:** 2017-02-04

**Authors:** Robert M. Badeau, Miikka-Juhani Honka, Marco Bucci, Patricia Iozzo, Johan G. Eriksson, Pirjo Nuutila

**Affiliations:** 1 Turku PET Centre, University of Turku, Turku FI-20521, Finland; mjhonk@utu.fi (M.-J.H.); marco.bucci@utu.fi (M.B.); patricia.iozzo@ifc.cnr.it (P.I.); pirjo.nuutila@utu.fi (P.N.); 2 Division of Natural Sciences, Department of Health Sciences, Franklin Pierce University, Rindge, NH 03461, USA; 3 Institute of Clinical Physiology, National Research Council, Pisa 56124, Italy; 4 Folkhälsan Research Centre, Helsinki FI-00250, Finland; johan.eriksson@helsinki.fi; 5 Department of Chronic Disease Prevention, National Institute for Health and Welfare, Helsinki FI-00271, Finland; 6 Department of General Practice and Primary Health Care, University of Helsinki and Helsinki University Hospital, Helsinki FI-00014, Finland; 7 Vaasa Central Hospital, Vaasa FI-65130, Finland; 8 Department of Endocrinology, Turku University Hospital, Turku FI-20521, Finland

**Keywords:** 22:6 docosahexaenoic acid, ω-3 fatty acids, offspring of obese mothers, offspring of lean mothers, insulin-dependent skeletal muscle glucose uptake

## Abstract

Background: Obesity among pregnant women is common, and their offspring are predisposed to obesity, insulin resistance, and diabetes. The circulating metabolites that are related to insulin resistance and are associated with this decreased tissue-specific uptake are unknown. Here, we assessed metabolite profiles in elderly women who were either female offspring from obese mothers (OOM) or offspring of lean mothers (OLM). Metabolic changes were tested for associations with metrics for insulin resistance. Methods: Thirty-seven elderly women were separated into elderly offspring from obese mothers (OOM; *n* = 17) and elderly offspring from lean/normal weight mothers (OLM; *n* = 20) groups. We measured plasma metabolites using proton nuclear magnetic resonance (^1^H-NMR) and insulin-dependent tissue-specific glucose uptake in skeletal muscle was assessed. Associations were made between metabolites and glucose uptake. Results: Compared to the OLM group, we found that the docosahexaenoic acid percentage of the total long-chain *n*-3 fatty acids (DHA/FA) was significantly lower in OOM (*p* = 0.015). DHA/FA associated significantly with skeletal muscle glucose uptake (GU) (*p* = 0.031) and the metabolizable glucose value derived from hyperinsulinemic-euglycemic clamp technique (M-value) in the OLM group only (*p* = 0.050). Conclusions: DHA/FA is associated with insulin-dependent skeletal muscle glucose uptake and this association is significantly weakened in the offspring of obese mothers.

## 1. Introduction

Globally, the incidence of obesity and subsequent insulin resistance and type 2 diabetes is rapidly rising. In North America, approximately 60% of women of reproductive age are obese [1], and, if current lifestyle habits continue, the global incidence of maternal obesity will increase [2]. Children from obese mothers have a significantly higher risk for developing these metabolic abnormalities than children from lean mothers [3,4]. Maternal obesity predisposes the offspring to insulin resistance starting in utero [5] through fetal programming [6]. As these children become elderly, the consequences of the innate developmental programming leading to insulin resistance and impaired tissue-specific glucose uptake remain unclear.

Elderly female offspring of obese mothers have decreased whole-body and muscle glucose uptake compared to elderly female offspring of lean mothers [6], and this suggests that the effects on insulin resistance due to maternal programming may endure over a lifetime.

Circulating metabolites can yield intriguing insights into the extent of insulin resistance. Also, the amounts of circulating metabolites may be indicative of hidden, intracellular mechanisms underlying this insulin resistance. In large epidemiological studies, branched-chained amino acid metabolites are associated with insulin resistance [7,8,9,10]. This indicates that obesity-induced insulin resistance causes profound changes in circulating metabolites and lipid profiles.

Here, we investigated the circulating metabolite profiles in elderly females who were the offspring of obese and non-obese women. Because the female offspring of obese mothers have significant skeletal muscle insulin resistance [11], we hypothesized that there should be a corresponding difference in circulating metabolites, and that these affected metabolites could be associated with tissue-specific insulin resistance.

## 2. Materials and Methods

### 2.1. Study Design and Participants

This study was derived from a completed investigation of a European Union–funded program called the Developmental ORIgins of healthy and unhealthy AgiNg (DORIAN) [11,12] and is registered at Clinicaltrials.gov as NCT01931540. It aimed to quantify how elderly offspring are affected by maternal frailty and degree of adiposity. Elderly women were selected from a large longitudinal birth cohort consisting of 13,345 individuals. These participants were selected from a randomly selected sub-cohort of 2003 individuals. Two groups were made according to maternal body to mass index (BMI) before delivery: the groups were called the offspring of overweight mothers (OOM) and the offspring of lean/normal weight mothers (OLM). The OOM group (*n* = 17) was selected according to maternal BMI ≥ 28.1 kg/m^2^ and the OLM group (*n* = 20) with a maternal BMI ≤ 26.3 kg/m^2^.

Participants who had diabetes requiring drug treatment or a fasting plasma value >7 mmol/L were excluded. Smokers or those with comorbidities that impact insulin sensitivity and contraindications for participating in exercise were excluded. All participants gave their written informed consent.

### 2.2. Metabolite Profiling

Fasting serum samples were stored in −70 °C. A high-throughput nuclear magnetic resonance (NMR) metabolomics platform was used for absolute quantification of circulating metabolites. The NMR-based metabolite profiling platform has previously been employed in various epidemiological studies and details of the experimentation have been described previously [10,13].

### 2.3. Measurement of Tissue-Specific Glucose Uptake

Positron emission tomography (PET) and computerized tomography (CT) analyses were performed as described in reference [11]. Briefly, the PET/CT study was performed after an overnight fast (12 h). Body fat percentage was measured using a bioelectrical impedance scale (Omron, model HBF-400-E, Kyoto, Japan). Two catheters were inserted: one into the antecubital vein of the right arm for saline infusion and blood sampling, another into the left arm for glucose, insulin, and fludeoxyglucose (^18^F-FDG) injection. After catheterisation, baseline blood samples were collected. The rate of insulin infusion was 1 mU/kg/min (Actrapid; Novo Nordisk, Copenhagen, Denmark). During hyperinsulinemia, normoglycemia was maintained by a variable infusion rate of 20% glucose based on plasma glucose determinations taken every 5–10 min from arterialized blood. After 40–50 min, when a steady state condition was reached, the subject was moved into the PET/CT scanner (Discovery 690, General Electric (GE) Medical systems, Milwaukee, WI, USA). ^18^F-FDG was administered and the scanning started immediately after tracer injection. The chest area was imaged first for 35 min (frames 8 × 15 s, 3 × 60 s, 6 × 300 s) to image the heart cavity, and the femoral area was scanned approximately 60 min after injection for 15 min (frames 5 × 180 s). CT scans were obtained in between the PET scans. The arm for blood sampling was warmed up with a heating pillow to arterialize venous blood. During the scan, arterialized venous blood samples were drawn for the determination of blood radioactivity, measured using an automatic gamma counter (“Wizard 1480 3”, Wallac, Turku, Finland). Insulin and glucose concentrations were measured at 0, 30, 60, and 90 min after the ^18^F-FDG injection.

### 2.4. PET/CT Data Processing

The software Carimas (v.2.71, Turku PET Centre, Turku, Finland) was used to analyze PET/CT images of the thighs. Image data were corrected for dead time, decay, and photon attenuation (based on CT images). Regions of interest (ROIs) were drawn manually on the images on every chosen skeletal muscle compartment (quadriceps, adductor magnus, hamstring, and adductor longus of both thighs of the participants. An anatomical reference on the CT images was adopted to draw on slices in the same position among subjects.

After obtaining the time activity curves (skeletal muscle and input function), kinetic modeling using the Gjedde-Patlak graphical method was performed to obtain the net influx rate (Ki) of FDG in skeletal muscle. Skeletal muscle glucose uptake per kg of tissue (GU) was calculated by multiplying Ki by the plasma glucose concentration and dividing by a lumped constant of 1.2. GU per depot (depot GU) was calculated as GU multiplied for the skeletal muscle group mass.

### 2.5. Statistics

All values are presented as means ± standard deviation (SD). Descriptive analysis and independent-samples t-tests were performed. For independent t-testing, if the variable did not fit a normal distribution, they were transformed by square root computations and analyzed. Correlations to tissue-specific glucose uptake were made to any metabolites that were found to be significantly different between the OOM and OLM groups. SPSS v.23 (IBM Corp. Released 2013. IBM SPSS Statistics for Windows, Version 23.0, Armonk, NY, USA) was used for statistical computations and graphs were drawn using GraphPad Prism (version 5.03 for Windows, GraphPad Software, La Jolla, CA, USA).

## 3. Results

**Figure 1:**
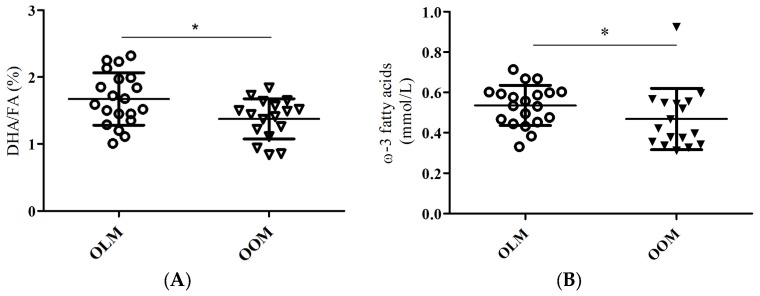
Differences in circulating docosahexaenoic acid to total fatty acid ratio (DHA/FA) percentages and ω-3 FA concentrations in female offspring from lean mothers (OLM) or female offspring from obese mothers (OOM). (**A**) Circulating DHA/FA percentages. * *p* = 0.015 between OLM and OOM groups; (**B**) Circulating ω-3 FA concentrations (FAw3). * *p* = 0.037 between OLM and OOM groups. Mean ± standard deviation (SD) are shown.

We separated the elderly participants into the OOM or OLM group based on their mother’s BMI (Table 1). We profiled circulating metabolites that are associated with insulin resistance or glucose metabolism between the OOM and OLM groups (Table 2 and Supplementary Table S1). The ratio of docosahexaenoic acid to total fatty acid percentages (DHA/FA) was significantly lower in the OOM group compared to the OLM group (Table 2, Figure 1A; *p* = 0.015). Also, the omega-3 fatty acids (FAw3) concentrations were significantly lower in the OOM group compared to the OLM group (Table 2, Figure 1B; *p* = 0.011).

**Figure 2:**
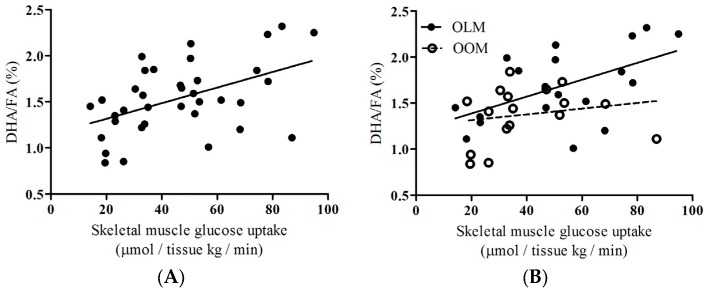
Correlations of DHA/FA ratios (percentages) to skeletal muscle glucose uptake between OOM and OLM groups. (**A**) In the whole group, Spearman’s *rho* value was 0.444 (*p* = 0.007). *n* = 36; (**B**) Spearman’s *rho* value for OLM group was 0.496 (*p* = 0.031) and for OOM group was −0.165. OLM: *n* = 19; OOM: *n* = 16.

**Figure 3:**
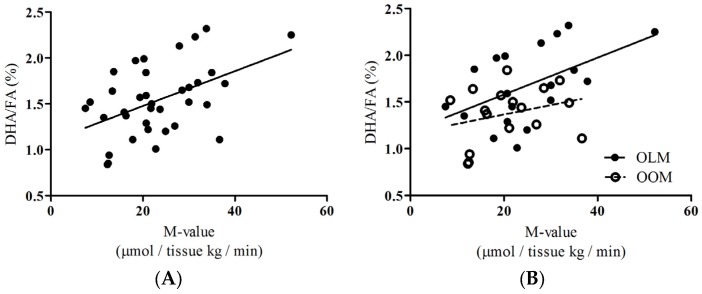
Correlations of DHA/FA ratios (percentages) to whole-body glucose uptake or, metabolizable glucose value derived from hyperinsulinemic-euglycemic clamp technique (M-value) between OOM and OLM groups. (**A**) Whole group. Spearman’s *rho* value was 0.396 (*p* = 0.017), *n* = 36; (**B**) Spearman’s *rho* value for OLM group was 0.457 (*p* = 0.050) and for OOM group was −0.124. OLM *n* = 19; OOM *n* = 16.

To provide insight about the group as a whole, we performed correlations of DHA/FA percentages to skeletal muscle glucose uptake (SKM-GU) and metabolizable glucose value derived from hyperinsulinemic-euglycemic clamp technique (M-values) and found that the whole group showed significant positive associations (Figure 2A: *rho* = 0.444, *p* = 0.007; Figure 3A: *rho* = 0.396, *p* = 0.017, respectively). We did not find any correlations between the SKM-GU and M-value with FAw3 concentrations in the whole group or between the OOM or OLM groups.

Insulin-dependent skeletal muscle glucose uptake, SKM-GU, and whole-body glucose uptake (expressed as the M-value) were our key outcome variables. DHA/FA correlated strongly with the SKM-GU (Figure 2B; *rho* = 0.496, *p* = 0.031) and M-value (Figure 3B; *rho* = 0.457, *p* = 0.050) in the OLM group, whereas DHA/FA did not correlate with the SKM-GU (Figure 2B) and M-value and the linear regression line was inverted in the OOM group (Figure 3B).

## 4. Discussion

This study was a substudy investigating the long-term impact of maternal obesity on aged offspring [11,12]. We tested the hypothesis that maternal obesity during pregnancy may predispose the elderly offspring to differences in circulating metabolites that associate with skeletal muscle glucose uptake. Our major findings are that, firstly, DHA/FA percentages and FAw3 concentrations were significantly different between the OOM and OLM groups (Table 2; Figure 1A,B, respectively). Secondly, a significant correlation existed between insulin-dependent skeletal muscle glucose uptake and DHA/FA percentages in the OLM group, but not in the OOM group (Figure 2B). The M-value association with DHA/FA was also significant in the OLM group, but not in the OOM group (Figure 3B).

Previously, we showed that in later life, female OOM are more insulin-resistant than OLM [11]. Here, we found that OOM have decreased DHA/FA percentages compared to OLM women (Figure 1). We also found that, in the OOM group, there was no statistical association between DHA/FA percentages and whole-body glucose uptake, whereas there were associations in the OLM group (Figure 3B). This suggests that the innate DHA metabolism may be impaired. Overall, the separate linear regression patterns found in the associations between DHA/FA and insulin-dependent glucose uptake and the significant differences in DHA/FA percentages found between the OLM and OOM groups imply that maternal obesity may predispose the offspring to impaired insulin sensitivity through DHA-related metabolic pathways.

DHA is a long-chain, polyunsaturated *n*-3 fatty acid commonly found in fish oil and is commonly taken as a dietary supplement. In humans, fish oil containing DHA prevents insulin resistance and glucose intolerance [14]. Our results support this line of evidence and highlight the role that DHA exclusively plays in mediating these preventative mechanisms. Our results showed that in the pooled group of women, there was a strong association between insulin-dependent skeletal muscle uptake and whole-body insulin sensitivity values and the DHA/FA percentage (Figure 3). In human cells, DHA has been proposed to inhibit the synthesis and secretion of cytokines involved with tumor necrosis factor (TNF)-α signaling [15]. In addition, *delta*-six and *delta*-five desaturases are important in the metabolism of long-chain polyunsaturated fatty acids, such as DHA. Caloric intake reduces the expression of these desaturases, resulting in decreased circulating levels of these fatty acids, including DHA [15]. Taken together, maternal obesity may invoke defects in multiple mechanisms that lead to decreased circulating DHA. This mechanism remains to be proven.

DHA/FA specifically affects insulin-dependent skeletal muscle glucose uptake. We did not find any correlations with other tissue-specific glucose uptake in the whole group or in the OOM and OLM groups when analyzed separately. We assessed insulin-dependent hepatic, subcutaneous fat, and myocardial glucose uptake. Also, we measured endogenous glucose production, and there was no association with this variable. During physiological hyperinsulinemia, skeletal muscle drives approximately 80% of glucose uptake in the body [16] and, therefore, these results were not surprising. However, this information is important as DHA is metabolized in the liver, and the liver produces endogenous glucose.

Maternal obesity did not predispose the offspring to changes in circulating branched-chain amino acids. A seminal report by Newgard et al. showed that circulating branched-chain amino acid concentrations are different between lean and obese humans and are markers for insulin resistance [7]. This was followed by numerous reports citing that circulating branched-chain amino acids associate with insulin resistance [8,9,10]. This discrepancy may be due to either the age of the study population or due to specific prenatal programming existing in the offspring of obese mothers.

Limitations to this study reside in the dietary habits and metabolomics methods. There is a possibility that the OOM group did not take DHA supplements or consumed lesser amounts of cold water fish, such as salmon, than the OLM group did. We did find that absolute levels of FAw3 were significantly reduced in the OOM group (Figure 1B). However, we consider this a remote possibility, because the women had a similar lifestyle and such a significant difference could only result in two very independent groups such as, for example, living in different regions of the world. In addition, absolute values of DHA were not significantly different between the groups. This rules out dietary-based confounding factors. Based on our data, it could be concluded that because of the lesser amount of absolute levels of FAw3 in the fatty acid milieu of the OOM women may affect DHA-mediated actions of insulin-dependent glucose uptake (Figure 1B). Additionally, we were limited by our method to quantitate the various fatty acid species present in serum. We could not ascertain the concentrations of circulating ceramide, and we could not quantify intracellular ceramide concentrations. Technically, we did not use LC/MS gas chromatography, which would be the appropriate method to determine ceramide and various species of diacylglycerol. Our technique, proton nuclear magnetic resonance spectroscopy (^1^H-NMR), profiles lipoprotein subclasses efficiently and general fatty acids and amino acids. We did not find, however, differences between the OOM and OLM group in overall diacylglycerides or sphingomyelin, a sphingolipid. Whether maternal obesity directly affects DHA through the lipid species existing in fatty acid milieu and other lipids implicated in insulin resistance remains uncertain.

## 5. Conclusions

In conclusion, we present original evidence that circulating DHA/FA percentages are positively associated with insulin-dependent skeletal muscle and whole-body glucose uptake in the offspring of lean mothers, but do not associate with these parameters in the offspring of obese mothers. This suggests that maternal obesity may impair DHA’s ability to participate in insulin metabolism and may contribute to insulin resistance.

## Figures and Tables

**Table 1:** Characteristics of study participants and of their mothers.

	OLM	OOM
*n*	20	17
Age	72 ± 2.6	71 ± 3.6
BMI (Mothers)	22.9 ± 1.4	29.7 * ± 1.6
BMI (Offspring)	26.6 ± 4.8	27.9 ± 4.57
Fasting plasma glucose (mmol/L)	5.99 ± 0.69	5.94 ± 0.77
Fasting insulin (mU/L)	9.60 ± 6.4	9.47 ± 4.59

Mean ± standard deviation (SD) are presented. Lean mothers (OLM) are the offspring of lean mothers and obese mothers (OOM) are the offspring of obese mothers. Mass index (BMI) is body to mass index expressed as height/km^2^. BMI of mothers is presented to define how obese mothers were at time of birth. Remaining values are only for their offspring. * *p* < 0.001. These data were published in [11], but the BMI values of the mothers have not been published.

**Table 2:** Circulating metabolites that are associated with insulin resistance and glucose metabolism.

	OOM	OLM
Variable	Mean ± SD	Mean ± SD
Total fatty acids	9.517 ± 1.500	9.353 ± 1.089
Docosahexaenoic acid	0.132 ± 0.043	0.155 ± 0.036
18:2, linoleic acid	2.561 ± 0.420	2.403 ± 0.327
Omega-3 fatty acids	0.468 ± 0.152	0.536 * ± 0.100
Omega-6 fatty acids	3.218 ± 0.492	3.121 ± 0.355
Saturated fatty acids	3.476 ± 0.554	3.373 ± 0.382
DHA/FA	1.376 ± 0.300	1.673 * ± 0.391
Alanine	0.364 ± 0.066	0.348 ± 0.035
Glutamine	0.506 ± 0.048	0.487 ± 0.047
Histidine	0.057 ± 0.009	0.055 ± 0.008
Isoleucine	0.045 ± 0.011	0.042 ± 0.011
Leucine	0.055 ± 0.012	0.055 ± 0.012
Valine	0.162 ± 0.032	0.164 ± 0.033
Phenylalanine	0.061 ± 0.008	0.062 ± 0.005
3-hydroxybutyrate	0.198 ± 0.144	0.183 ± 0.129
a1-acid glycoprotein	1.114 ± 0.110	1.082 ± 0.114

Data are expressed as mean ± SD. Metabolites are expressed in mmol/L except where indicated otherwise. DHA/FA represents the docosahexaenoic acid to total fatty acid ratio. * *p* < 0.05. OOM *n* = 15; OLM *n* = 20. DHA/FA: Ratio of 22:6 docosahexaenoic acid to total fatty acids.

## Supplementary Materials

The following are available online at http://www.mdpi.com/2072-6643/9/2/110/s1, Table S1: Circulating fatty acids and metabolites in offspring from obese mothers (OOM) and the offspring of lean mothers (OLM).

## Abbreviations

BMI, body to mass index; DHA, docosahexaenoic acid; FA, fatty acids; ^18^F-FDG, fludeoxyglucose; GU, glucose uptake; ^1^H-NMR, proton nuclear magnetic resonance; M-value, metabolizable glucose value derived from hyperinsulinemic-euglycemic clamp technique; OLM, offspring from lean mothers; OOM, offspring from obese mothers; PET, positron-emission tomography; SD, standard deviation; SKM, skeletal muscle; TNF, tumor necrosis factor.
