# Supplementary material for: Circulating Docosahexaenoic Acid Associates with Insulin-Dependent Skeletal Muscle and Whole Body Glucose Uptake in Older Women Born from Normal Weight Mothers
Source: Nutrients. 2017 Feb 4;9(2):110. doi: 10.3390/nu9020110 (PMC5331541; doi:10.3390/nu9020110)
Supplement: Supplementary file 1 [file nutrients-09-00110-s001.docx]

Supplementary Materials: Circulating Docosahexaenoic Acid Associates with Insulin-Dependent Skeletal Muscle and Whole Body Glucose Uptake in Older Women Born from Normal Weight Mothers

Robert M. Badeau, Miikka-Juhani Honka, Marco Bucci, Patricia Iozzo, Johan G. Eriksson and Pirjo Nuutila

**Table S1.** Circulating fatty acids and metabolites in offspring from obese mothers (OOM) and the offspring of lean mothers (OLM).

|  | **OOM** | **OLM** |
| --- | --- | --- |
| Variable | Mean ± SD | Mean ± SD |
| Total fatty acids | 9.517 ± 1.500 | 9.353 ± 1.089 |
| 22:6, docosahexaenoic acid | 0.132 ± 0.043 | 0.155 ± 0.036 |
| 18:2, linoleic acid | 2.561 ± 0.420 | 2.403 ± 0.327 |
| Omega-3 fatty acids | 0.468 ± 0.152 | 0.536 * ± 0.100 |
| Omega-6 fatty acids | 3.218 ± 0.492 | 3.121 ± 0.355 |
| Saturated fatty acids | 3.476 ± 0.554 | 3.373 ± 0.382 |
| DHA/FA (Ratio of 22:6 docosahexaenoic acid to total fatty acids) | 1.376 ± 0.300 | 1.673 * ± 0.391 |
| Ratio of 18:2 linoleic acid to total fatty acids | 27.00 ± 2.801 | 25.72 ± 2.346 |
| Ratio of conjugated linoleic acid to total fatty acids | 0.069 ± 0.054 | 0.099 ± 0.088 |
| Ratio of omega-3 fatty acids to total fatty acids | 4.855 ± 0.953 | 5.763 ± 1.111 |
| Ratio of omega-6 fatty acids to total fatty acids | 33.89 ± 2.319 | 33.44 ± 2.110 |
| Ratio of polyunsaturated fatty acids to total fatty acids | 38.74 ± 2.503 | 39.19 ± 2.234 |
| Ratio of monounsaturated fatty acids to total fatty acids | 24.75 ± 2.653 | 24.72 ± 2.966 |
| Ratio of saturated fatty acids to total fatty acids | 36.51 ± 1.353 | 36.10 ± 1.738 |
| Glucose | 4.418 ± 0.471 | 4.369 ± 0.543 |
| Lactate | 1.315 ± 0.234 | 1.222 ± 0.200 |
| Pyruvate | 0.074 ± 0.022 | 0.070 ± 0.014 |
| Citrate | 0.117 ± 0.016 | 0.112 ± 0.018 |
| Glycerol | 0.084 ± 0.032 | 0.081 ± 0.034 |
| Alanine | 0.364 ± 0.066 | 0.348 ± 0.035 |
| Glutamine | 0.506 ± 0.048 | 0.487 ± 0.047 |
| Glycine | 0.264 ± 0.046 | 0.260 ± 0.072 |
| Histidine | 0.057 ± 0.009 | 0.055 ± 0.008 |
| Isoleucine | 0.045 ± 0.011 | 0.042 ± 0.011 |
| Leucine | 0.055 ± 0.012 | 0.055 ± 0.012 |
| Valine | 0.162 ± 0.032 | 0.164 ± 0.033 |
| Phenylalanine | 0.061 ± 0.008 | 0.062 ± 0.005 |
| Tyrosine | 0.050 ± 0.008 | 0.050 ± 0.008 |
| Acetate | 0.041 ± 0.007 | 0.041 ± 0.008 |
| Acetoacetate | 0.094 ± 0.070 | 0.079 ± 0.048 |
| 3-hydroxybutyrate | 0.198 ± 0.144 | 0.183 ± 0.129 |
| Creatinine | 0.053 ± 0.009 | 0.053 ± 0.010 |
| Albumin | 0.076 ± 0.005 | 0.077 ± 0.004 |
| Glycoprotein acetyls, mainly a1-acid glycoprotein | 1.114 ± 0.110 | 1.082 ± 0.114 |

Data are expressed as mean ± standard deviation (SD). Metabolites are expressed in mmol/L except where indicated otherwise. Ratios are expressed as percentages. * *p* < 0.05 comparing OOM and OLM. OOM *n* = 15, OLM *n* = 20.
